# Touching with the eyes: Oculomotor self-touch induces illusory body ownership

**DOI:** 10.1016/j.isci.2023.106180

**Published:** 2023-02-10

**Authors:** Antonio Cataldo, Massimiliano Di Luca, Ophelia Deroy, Vincent Hayward

**Affiliations:** 1 Institute of Philosophy, School of Advanced Study, University of London, Senate House, London WC1E 7HU, UK; 2 Cognition, Values and Behaviour, Ludwig Maximilian University, 80333 München, Germany; 3 Institute of Cognitive Neuroscience, University College London, Alexandra House 17 Queen Square, London WC1N 3AZ, UK; 4 Formerly with Facebook Reality Labs, Redmond, WA, USA; 5 School of Psychology, University of Birmingham, Edgbaston, Birmingham B15 2TT, UK; 6 Institut des Systèmes Intelligents et de Robotique, Sorbonne Université, 75005 Paris, France

**Keywords:** Cognitive neuroscience, Sensory neuroscience, Techniques in neuroscience

## Abstract

Self-touch plays a central role in the construction and plasticity of the bodily self. But which mechanisms support this role? Previous accounts emphasize the convergence of proprioceptive and tactile signals from the touching and the touched body parts. Here, we hypothesise that proprioceptive information is not necessary for self-touch modulation of body-ownership. Because eye movements do not rely on proprioceptive signals as limb movements do, we developed a novel oculomotor self-touch paradigm where voluntary eye movements generated corresponding tactile sensations. We then compared the effectiveness of eye versus hand self-touch movements in generating an illusion of owning a rubber hand. Voluntary oculomotor self-touch was as effective as hand-driven self-touch, suggesting that proprioception does not contribute to body ownership during self-touch. Self-touch may contribute to a unified sense of bodily self by binding voluntary actions toward our own body with their tactile consequences.

## Highlights

• The mechanisms linking voluntary self-touch to bodily self-awareness are unclear
• We used an oculo-tactile paradigm to test the role of proprioception in self-touch
• Proprioception was not necessary to induce body ownership over a rubber hand
• Bodily self-awareness may arise from the binding properties of voluntary self-touch

## Graphical abstract

**Figure:**
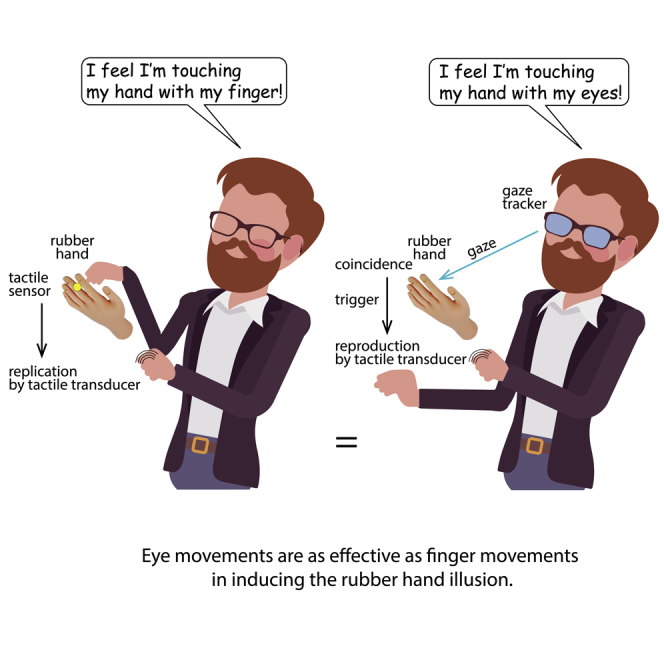

## Introduction

Touching our hand, by contrast with that of a stranger, immediately tells us that it is our own. Hand-to-body movements elicit some of the earliest and ubiquitous sensorimotor experiences in life, and have already taken place *in utero*.^1^ These experiences continue throughout the entire lifespan^2^ with a frequency of up to 800 self-touches a day.^3^ Self-touch is arguably one of the richest sensorimotor experiences, providing a tight contingency between motor, proprioceptive, and tactile information from both the touching and the touched body parts. Since the mid-20th century,^4^ the unique sensorimotor contingency typical of self-touch has been linked with the development of self-awareness and body ownership.^5^^,^^6^^,^^7^^,^^8^^,^^9^^,^^10^^,^^11^ Yet, the specific mechanisms behind the binding of self-touching actions with inner and outer sensations remain elusive. Earlier accounts stress the importance of the unique convergence of proprioceptive information generated by the moving effector and the tactile signals arising from the touched body region.^4^^,^^5^^,^^6^^,^^12^^,^^13^ To the best of our knowledge, however, no study has directly investigated whether proprioception is a necessary for self-touch to modulate body ownership. The voluntary nature of self-touching movements might, in fact, be sufficient to explain the binding properties of self-touch.^11^^,^^14^^,^^15^^,^^16^^,^^17^ For example, Hara et al. found that voluntary self-touch boosts the sense of body ownership compared to passive self-touch and classical multisensory stimulation.^11^

**Figure 1:**
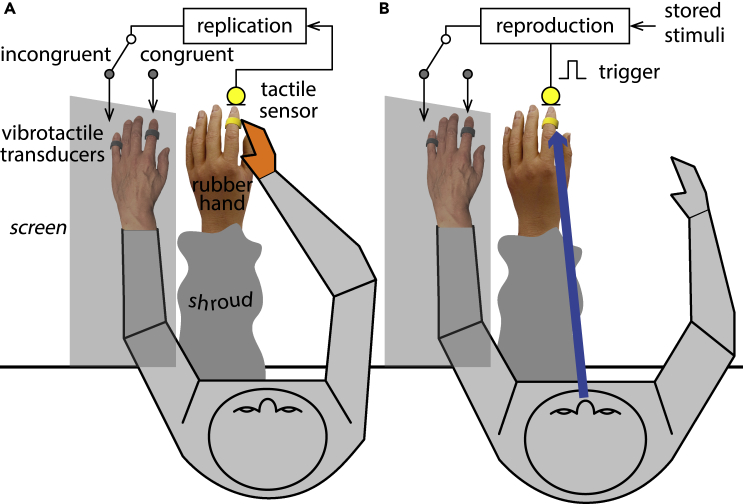
Experimental conditions (A) In the hand-driven “proprioception” condition, participants’ touch of the index finger of a rubber hand was replicated on either their left index (congruent) or little finger (incongruent) (see Video S1). (B) In the eye-driven “no-proprioception” condition, participants’ gaze on the index finger of the rubber hand triggered the reproduction of a touch on either their left index (congruent) or little finger (incongruent) (see Video S2). In both effector conditions, participants reported the onset time of the illusion, then performed a crossmodal congruency task, and finally answered a self-report questionnaire. See STAR Methods and Figures S1, S4, and S5.

Disentangling the role of afferent proprioception from that of efferent motor signals in self-touch is difficult because self-touch normally involves voluntary limb movements, thus always generating proprioceptive signals from the moving effector. Here, we dissociated proprioception from other motor signals by creating a self-touch condition where tactile sensations were linked to voluntary eye movements. Eye movements do not depend on proprioceptive information as limb movements do.^18^ It is known that the absence of proprioceptive afferent signals severely impairs limb movements, as demonstrated by studies with deafferented patients.^19^ Conversely, lesions to the oculomotor system in monkeys^20^ and in humans^18^ do not impair the accuracy and precision of gaze pointing, suggesting that eye proprioception does not participate in visual localization.^21^ Thus, in an artificially created oculomotor self-touch condition, the proprio-tactile convergence hypothesis would predict that eye movements would weaken an experience of body ownership, compared to ordinary, hand-driven, self-touch. We tested this hypothesis by comparing the effectiveness of hand versus eye movements in eliciting the so-called *rubber hand illusion* (RHI).^22^ We used a 2 (effector type: hand, eyes) by 2 (spatial congruency: congruent, incongruent) within-participants experimental design to test a hand-driven self-touch condition (Figures 1A and S1A) against an eye-driven self-touch condition (Figure 1B). We collected subjective measures of self-reports and time of onset of the RHI^22^^,^^23^ and objective measures of cross-modal congruency task^24^ of the occurrence of RHI.

We expected a significant effect of spatial congruency in all tested measures.^22^^,^^25^ Crucially, if the afferent proprioceptive signals from the moving effector were not necessary for body ownership, then hand and eye self-touch movements should have been equally effective in eliciting the illusion. The experiment was designed to test the null hypothesis, i.e., there was no difference between eye and hand self-touch conditions. To this end, Bayesian analyses^26^^,^^27^ were used to test the lack of significant interaction between main factors. Participants performed four blocks, one for each condition of our 2 by 2 experimental design. Each block started with a 2-min hand/eye-driven self-touch stimulation, during which participants reported the onset time of the illusion; then a crossmodal congruency task took place for about 12 min; finally, participants responded to the self-report questionnaire (see Figure S1B).

## Results

During the self-stimulation phase, participants viewed a rubber hand located in the vicinity of their occluded left hand. In the hand-driven “proprioception” condition (Figure 1A), participants reached out and touched the index finger of a left rubber hand with their right hand. The impact was captured by a sensor concealed inside the rubber finger and was replicated by one of two vibrotactile transducers, stimulating either the participants’ left index (congruent condition) or the little finger (incongruent condition) (see Video S1). In the eye-driven “no-proprioception” condition (Figure 1B), participants first fixated a mark in the vicinity of their resting right hand and then voluntarily directed their gaze to the index finger of the rubber hand. An eye-tracker monitored the participant’s gaze. Gazing on the index finger of the rubber hand triggered the reproduction of pre-recorded, realistic touch sensations on either the participants’ left index (congruent condition) or little finger (incongruent condition) (see Video S2). To match the vision of the moving finger in the hand-driven condition, the participants’ gaze was continuously indicated by a red dot projected on the table. Thus, the two self-touch conditions were identical in all respects apart from the effector used to perform the self-touching movements. The eye-driven “no-proprioception” condition provided comparable motor commands but with much weaker proprioceptive information than the hand movement condition.^18^

Video S1. Experimental setup for the Haptic Rubber Hand Illusion Video depicting the experimental setup for the hand-driven self-touch condition related to Figure 1.

Video S2. Experimental setup and procedure Video depicting the experimental setup, procedure, and illustrative trials of the hand-driven and oculomotor self-touch conditions related to Figure 1.

During the self-stimulation phase, participants were asked to report the onset time of the rubber hand illusion, with shorter onset times indicating a stronger illusion.^23^ Participants then performed a cross-modal congruency task (CCT).^24^ They identified the location of tactile stimuli delivered to either their left index or middle finger, while ignoring visual distractors projected on the fingers of the rubber hand. The task comprised thirty homologous and thirty non-homologous trials. Longer response times to non-homologous trials indicated a stronger interference between visual and tactile stimuli, and therefore a stronger illusion.^24^ Finally, participants completed a subjective report, answering three questions on the strength of the illusion and four control questions.^22^ See STAR Methods.

**Figure 2:**
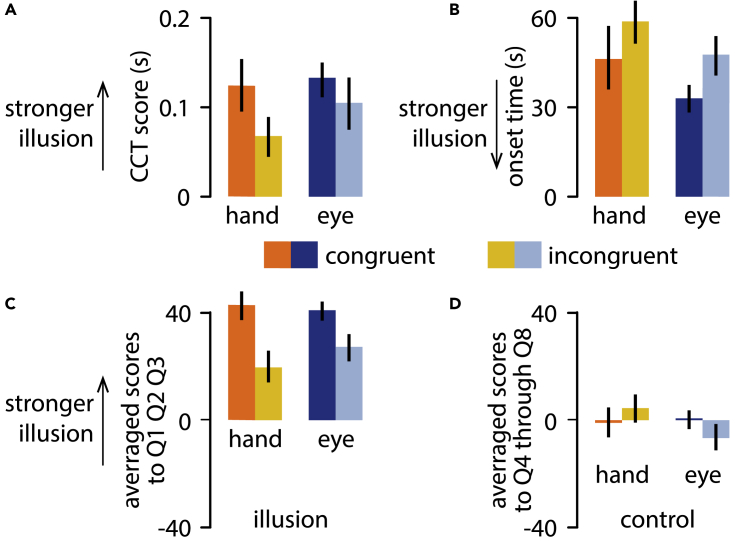
Results (A) CCT Scores. (B) Onset time. (C) Self-reports measures. In all tested measures and in both effector conditions, the illusion was significantly stronger when the tactile stimulus was spatially congruent with the location of touch/gaze on the rubber hand. Importantly, the difference between spatially congruent and incongruent conditions was similar between hand- and eye-driven conditions, as confirmed by a series of Bayesian t-test. (D) Responses to the control items of the self-report questionnaire did not show any significant effect. Error bars in each graph represent the standard error of the mean. See also STAR Methods, Figures S2 and S3.

All tested measures (CCT scores, onset time, and subjective reports) differed significantly according to the main effect of spatial congruency. A series of 2 by 2 repeated measures ANOVAs showed a significant main effect of spatial congruency (CCT: F_1,23_ = 5.17, p = 0.033, η_p_^2^ = 0.18; onset time: F_1,11_ = 8.33, p = 0.015, η_p_^2^ = 0.43; subjective report: F_1,19_ = 4.63, p = 0.044, η_p_^2^ = 0.20). In each tested measure, the illusion was significantly stronger in the spatially congruent condition (CCT: mean ±95 %CI: 128.6 ± 42.0; onset time: 39.7 ± 13 s; self-reports: 20.6 ± 12.7), compared to the spatially incongruent condition (CCT: 86.5 ± 44.7; onset time: 53.3 ± 12.3 s; self-reports: 10.7 ± 14.1) (see Figures 2A–2C, S2, and S3).

Crucially, the differences between spatially congruent and incongruent conditions were of equal magnitude in the “proprioception” and in the “no-proprioception” self-touch conditions, as there was no main effect of effector-type (CCT: F_1,23_ = 2.17, p = 0.154, η_p_^2^ = 0.09; onset time: F_1,11_ = 2.14, p = 0.172, η_p_^2^ = 0.14; subjective report: F_1,19_ = 0.01, p = 0.923, η_p_^2^< 0.01) nor interaction between factors (CCT: F_1,23_ = 0.62, p = 0.439, η_p_^2^ = 0.03; onset time: F_1,11_ = 0.04, p = 0.852, η_p_^2^< 0.01; subjective report: F_1,19_< 0.01, p = 0.991, η_p_^2^< 0.01) for each tested measure. A series of Bayesian t-tests^26^^,^^27^ on each dependent variable supported the null hypothesis of no difference between eye and hand movements for each measure (CCT: BF_01_ = 3.52, %error = 0.04; onset time: BF_01_ = 3.43, %error = 0.02; subjective report: BF_01_ = 3.13, % error = 0.02).

## Discussion

Self-touch plays a fundamental role in developing self-awareness and a sense of body ownership. Although previous studies have investigated the contribution of the tactile component of self-touch to self-representation,^5^ the present study focused on the motor component of self-touch. Specifically, we tested whether proprioceptive information arising from the moving effector is strictly necessary for self-touch to modulate body ownership. Eye movements do not crucially rely on afferent proprioceptive signals as limb movements do^18^ and eye movements do not typically produce tactile consequences. Despite the profound differences between eye movements and limb movements, coupling voluntary eye movements to spatially and temporally congruent tactile inputs induced an illusion of limb ownership as strongly and quickly as binding tactile inputs to limb movements. This effect was reliable across subjective and objective measures of body ownership and occurred without any preliminary training or habituation.

The present findings undermine earlier accounts of self-touch highlighting the contribution of a required convergence of tactile and proprioceptive signals.^4^^,^^12^ Our results align with recent studies suggesting that active self-touch movements enhance body ownership compared to passive movements and play a key role in the spatial coherence of bodily self-awareness.^14^^,^^28^ Yet, most of the previous studies on self-touch employed an active-versus-passive movements paradigm. Because proprioceptive signals were equally present in active and passive movements, those studies could not conclusively rule out that proprioception contributed to modulate body ownership through self-touching movements. Our findings, based on a newly introduced oculomotor self-touch paradigm, suggest that the convergence of proprio-tactile signals from the touching and the touched body parts on the sense of body ownership is only a special case of a more general mechanism.

Voluntary motor signals other than proprioception, such as motor imagery,^29^ may shape the experience of body ownership because they bind motor commands to tactile outcomes, leading to a unified experience of a single acting and perceiving self.^16^^,^^17^^,^^30^ In much the same way that turning a switch provides us with a sense of agency over the lights,^15^ active self-touch on our body may provide us with a sense of bodily self. Future studies should identify the specific motor component responsible for the observed effects, by directly testing the role of the sense of agency,^15^ efference copy,^31^ motor imagery,^29^ and causal inference.^17^^,^^32^

Sensorimotor accounts of the visual experience posit that sensorimotor contingencies linking efferent oculomotor signals to afferent visual feedback are learned from specific oculo-visual invariances.^33^ In ordinary conditions, eye movements affect visual inputs only, whereas reaching hand movements produce tactile feedback. A crucial prediction made by the sensorimotor theories is that an agent provided with new contingencies between movements and sensory outcomes should be able to learn new sensorimotor invariances and perceive through them. Studies on sensory substitution support this hypothesis.^34^^,^^35^^,^^36^ Our study shows that the artificial coupling of voluntary eye movements with tactile feedback produces a sensorimotor contingency that affects bodily self-awareness.

The oculomotor system is thought to participate in the spatial re-mapping of visual, auditory, and tactile inputs. Animal studies show that the superior colliculus provides the neural substrate for controlling voluntary saccades and integrating visual, auditory, and tactile stimuli into a common reference frame.^37^ Our finding may extend the scope of this mechanism by including the possibility that oculo-tactile spatial transformations can also modulate high-level cognitive processes such as bodily self-awareness, similarly to what it has previously been suggested for ordinary hand-driven sensorimotor experiences.^16^

Humans automatically look at what they are about to touch,^38^ but oculomotor and tactile events are not normally linked by a causal relationship since moving the eyes only affects visual inputs. The artificial coupling of tactile feedback in response to oculomotor signals in our experiment might have putatively leveraged a proactive function of eye movements^38^ to induce an instrumental use of the gaze. Updating body-ownership by binding self-generated gazing toward our own body to tactile consequences may find applications in future prosthetic devices, virtual reality, and human-machine interaction techniques.^39^

### Limitations of the study

The present experiment tested hand versus eye movements only under active (i.e., voluntary) conditions. Therefore, our data cannot quantify any potential difference between illusory body ownership induced by passive-versus-active movements. This choice was owed to the methodological impossibility of creating a passive eye movement condition. However, direct comparison of active-versus-passive movements has already been extensively investigated in previous studies.^8^^,^^11^^,^^14^^,^^28^^,^^40^^,^^41^ Moreover, the present study did not aim to investigate voluntary movements *per se*. Instead, it was aimed to test whether proprioceptive signals during self-touch were strictly necessary to modulate body ownership.

The present study focused on the role of proprioception in self-touch but could not quantify the contribution of tactile sensations, which were already investigated in previous experiments.^5^^,^^14^^,^^28^ Given that we used near-identical tactile stimuli in each condition, our results suggest that the effect of self-touch on body ownership goes over and above any potential effect of tactile stimulation. That is, the mere presence of a tactile stimulus coupled with a voluntary movement seems insufficient to modulate body-ownership, unless the spatial location of touch is also congruent with the location specified by the eye/hand motor plan.

Both our hand-driven and eye-driven self-touch conditions included some visual information (i.e., the vision of the moving finger and a red dot showing the participant’s gaze, respectively, plus the rubber hand). Thus, the present experiment cannot rule out the possibility of a three-way interaction between motor, tactile, and visual signals. First, participants’ eye movements were unconstrained during the hand-driven “proprioceptive” condition. Therefore, oculomotor (and visual) signals might have potentially contributed to the RHI in that condition. Future studies could rule out this possibility by investigating hand movements in the absence of vision (e.g., in darkness), or eye movements (e.g., by asking the participants to fixate on a specific point). However, performing blind hand movements toward a rubber hand would require extensive training, whereas we wanted to test untrained sensorimotor integration. Moreover, although blind hand movements are technically possible, equivalent no-vision eye movements are unfeasible, as voluntary saccades are highly inaccurate in darkness.^42^ Crucially, the eye movements in our hand-driven “proprioception” condition were only incidental, whereas those in our eye-driven “no-proprioception” condition had a clear instrumental effect, directly causing the tactile sensations experienced by the participant. Thus, an eventual role of eye movements in our “proprioception” condition would only make our findings in the “no-proprioception” condition more striking, by showing that the summed effect of hand movements plus incidental eye movements can be equalized by instrumental eye movements only.

Besides eye movements, the specific visual inputs present in both our conditions might affect the strength of the RHI. In our eye-driven “no-proprioception” condition, we used a red dot to continuously show the participants’ gaze throughout the block. This visual cue was necessary to enhance the internal validity of our study by ensuring that our eye-driven “no-proprioception” condition was visually matched to the hand-driven “proprioception” condition, where the participants could see their own hand performing the self-touch movements. Yet, previous studies have shown that it is possible to induce an RHI simply by creating a multisensory conflict matching a visual with a tactile stimulus,^43^^,^^44^ therefore, one may ask how specific our result is for sensorimotor, oculotactile integration as opposed to simple multisensory integration. Eye movements in our “no-proprioception” condition have a clear instrumental effect, with the participants’ saccades directly causing them to experience tactile sensations. That is, our artificial oculotactile coupling preserved the voluntary aspects of self-touch in that the participant experienced no tactile sensations if they decide not to perform a voluntary to saccade toward the rubber hand. In other words, compared to a classical visuotactile RHI stimulation, our condition necessarily involved a voluntary motor component akin to motor programming, efferent copy, and sense of agency. Although we did not collect any direct measure of the sense of agency for the visual cue in this study, all participants were able to internalize and utilize this new sensorimotor contingency after just a few seconds. In fact, the rapidity and effectiveness of our paradigm to induce a strong sense of control over a completely new oculotactile contingency is in itself an interesting result. Future studies directly address this question by investigating the role of sense of agency,^15^ efference copy,^31^ motor imagery,^29^ and causal inference^17^^,^^32^ in our paradigm.

Finally, it is known that visual signals that are incongruent with the representation of the body (e.g., seeing a block of wood instead of the rubber hand, or aligning the rubber hand in an anatomically incongruent position) weaken or abolish the RHI. Moreover, recent evidence shows that visual input can decrease the sensitivity of muscle proprioceptive feedback.^45^ Future studies should specifically address the interaction between self-touch and vision, for example by testing self-touch for body parts that are commonly on sight (e.g., forearms) vs parts of the body that are rarely seen (e.g., back). Moreover, future studies could disentangle the role of visual stimuli from that of voluntary oculomotor signals, for example by replicating our eye driven RHI condition for an unseen rubber hand.

Our active rubber hand illusion paradigm has limitations that should be addressed in future studies. The classical control condition for the active rubber hand illusion introduces a short delay between the movement and the tactile sensation. In the present study, however, we only investigated the effect of spatial congruency of touch and oculomotor signals. The reason for this choice was that the hand and the eye motor systems have vastly different temporal properties, with eye movements being orders of magnitude faster than any other human movement.^46^ Moreover, eye movements have a distinctly proactive nature, systematically anticipating hand-object contact by approximately ∼150 ms.^38^ Given that crossmodal integration is affected by the latencies of the signals involved,^37^ we surmised that oculotactile integration may have very different temporal properties than sensorimotor integration during hand movements. Future studies should directly address this question by testing the temporal dynamics of oculotactile versus limb sensorimotor integration. Nonetheless, the main effect of spatial congruency we found across all our dependent measures suggests that our spatial manipulation was as successful as the classical temporal manipulation in inducing different degrees of RHI. Second, besides self-reports, onset time, and CCT task, several other behavioral and physiological measures have been previously used to test the RHI, including the proprioceptive drift, skin conductance, skin temperature, and self-recognition.^25^ In particular, given that the proprioceptive drift (i.e., the perceived displacement of the left hand toward the location of the rubber hand) was shown to be independent from sensation of ownership,^47^ future experiments could test whether drift also occurs in gaze-contingent conditions similar to the one we developed.

Finally, previous studies have shown that simply seeing an object approaching (but not touching) a rubber hand located nearby the real hand can elicit a sensation of ownership over the rubber hand.^48^ Although these findings generally show that top-down predictions play an important role in the RHI, eye movements do not normally generate tactile feedback as hand movements do. Therefore, one may speculate that the participants’ prior expectation of a tactile stimulus would be much lower during our oculomotor self-touch condition than during normal, hand-driven self-touch conditions. Yet, we found strong RHI modulation after oculotactile stimulation. This result suggests either that our effect does not rely on the expectation of a tactile stimulus, or, alternatively, that such expectations are not disrupted by an artificial gaze-contingent tactile stimulation. Recent studies on the peripersonal space show that new statistical regularities can be quickly learnt, and new predictions can be created. For instance, pairing a visual (or auditory) stimulus with a tactile one can readily create new multisensory associations,^49^^,^^50^ sometimes based on very rapid trial-by-trial recalibration.^51^ Our results are in line with these findings and suggest that a new artificial oculotactile contingency can readily be learnt even if it involves self-touch and occurs on one’s own body rather than in the peripersonal space.

## STAR★Methods

### Key resources table

**Table:** 

REAGENT or RESOURCE	SOURCE	IDENTIFIER
**Deposited data**		

Raw and analyzed data	This paper and Supplemental Information	This paper and Supplemental Information

**Software and algorithms**		

MATLAB 2018a	MathWorks	https://www.mathworks.com/products/matlab.html
Psychophysics Toolbox v3	MathWorks	http://psychtoolbox.org
Arduino IDE 1.8.19	Arduino	https://www.arduino.cc/en/software
SPSS Statistics for Windows, version 23	IBM Corp	https://www.ibm.com/analytics/spss-statistics-software

**Other**		

Eyelink 1000+	SR Research	https://www.sr-research.com/eyelink-1000-plus
Haptuator Mark II	Tactile Labs Inc	http://tactilelabs.com/products/haptics/haptuator-mark-ii-v2
Cosmetic glove RPL 503/505	Realistic Prosthetics Ltd	http://www.realisticprosthetics.com

### Resource availability

#### Lead contact

Further information and requests for resources should be directed to and will be fulfilled by the lead contact, Vincent Hayward (vincent.hayward@sorbonne-universite.fr).

#### Materials availability

This study did not generate new unique material.

### Experimental model and subject details

#### Participants

The sample size of the study (n = 24; 16 females; mean age ±SD: 23.1 ± 2.7) was decided a-priori on the basis of the results of previous Crossmodal Congruency Task (CCT) experiments used to quantify the strength of the RHI.^52^ Twenty-eight right-handed healthy participants were originally recruited. One participant was excluded before the analyses due to technical issues with the tactile stimulation. Another participant was excluded from the analyses because they could not follow the instructions. Conforming with the standard procedure for CCT paradigms,^24^^,^^52^ two further participants were excluded after visual inspection of the data because they produced more than 35% false alarms in the catch trials of at least one experimental condition (see below).

The experiment was run in accordance with the protocol approved by the research ethics committee of the School of Advanced Study, University of London. Recruitment and procedures adhere to the Declaration of Helsinki. All participants were naïve regarding the hypotheses underlying the experiment. All participants received written and verbal explanation of the general purpose of the study, provided their written informed consent before the beginning of the testing, and were compensated for their time at a rate of £7.5 per hour (∼£10).

### Method details

#### General setup

Figure S1A shows a schematic representation of the experimental setup. Participants sat at a desk, resting their left arm on a 30° table easel (Granthams Ltd., Danube A2, UK) and their right arm on an articulated armrest support (YANGHX, model 4328350928, China). Head movements were constrained using a chin and forehead rest (SR Research, Canada). Participants’ view of their left arm and shoulder was blocked by a fixed vertical cardboard screen throughout the entire experiment. A left cosmetic silicone glove (Realistic Prosthetics Ltd., model RPL 503/505, UK) filled with cotton wool was placed in front of the participants, at about 15cm to the right of their left hand. A male or a female glove was used, according to the gender of each participant. A black gown was used to cover both the participants’ left arm and the cosmetic glove forearm up to the wrist.

Instructions and visual stimuli were projected onto the table easel and the rubber hand using an LCD projector (Nec, model LT150, Tokyo) mounted above the desk. Participants’ responses in different tasks were collected using either a mouse or a foot-pedal. Participants’ eye movements were recorded via an EyeLink 1000 Plus eye tracker (SR Research, Canada) arranged in a tower-mount configuration. Participantswore wireless headphones (Cowin Electronics Co. Ltd., model E7, China) playing pink noise throughout the entire experiment to mask any eventual noise from the tactile actuators. All the software for the experimental tasks were coded in Matlab 2018a (The MathWorks, Inc.,USA) and Psychophysics Toolbox v3.^53^^,^^54^

#### Setup for the hand movement condition

In the classical RHI, a trained experimenter uses two brushes to stroke homologous points on the participant’s and the rubber hand in sync, producing a passive, visuo-tactile stimulation.^22^ Voluntary self-touch versions of the RHI using virtual reality^55^ or robotic mediation^11^ have been also described. Here, however, we used an electronic circuit involving accelerometers and vibrotactile actuators to achieve a direct, unmediated haptic touch of the rubber hand (see the left panel in Figure S1A and Video S1).

In this “haptic” version of the RHI, participants used their own right index finger to directly tap on the index finger of the rubber hand and received a simultaneous and proportional mechanical touch on their own left hand, either on the same (index) or a different (little) finger. Tactile stimuli delivered on the rubber hand were detected by an accelerometer (SparkFun Electronics, model ADXL335, USA) located inside the index finger of the cosmetic glove, at the level of the intermediate phalanx. The analog output signal from the z-axis of the accelerometer was amplified (Shenzhen Cavins Technology Co., Ltd., Nobsound 50Wx2 Amplifier, China) to drive a vibrotactile actuator (Tactile Labs Inc., Haptuator Mark II, Canada) secured to the participants’ left index finger with a Velcro strip. In this setup, the vibrotactile pattern produced by the participant’s tap on the index finger of the rubber hand is instantaneously and faithfully reproduced on the congruent (index) finger of the participants’ left hand. A second actuator was attached to the participants’ little finger to create a spatially incongruent condition. An additional actuator was placed on the middle finger to perform the CCT task (see below). The appropriate actuator in each task and in each trial was activated by a computer-controlled switch. As the RHI is affected by the crossmodal correspondence of visuo-tactile stimuli,^25^ Velcro strips were also placed on the intermediate phalanxes of the index, middle, and little fingers of the cosmetic glove, providing a visual matching for the sensation produced by the strips holding the actuators on the participants’ fingers.

#### Experimental design

To compare the efficacy of hand and eye movements in inducing the RHI, we employed a 2 (effector-type: hand, eye) x 2 (spatial-congruency: congruent, incongruent) within-subject design. The effector-type factor was blocked and counterbalanced across participants, whilethe order of the spatial-congruency conditions was randomised within participant. To assess the strength of the RHI, we selected three of the best-established measures for RHI^25^ that are thought to underly different aspects of the illusion.^47^ First, the Crossmodal Congruency Task (CCT) is considered one of the most objective behavioural measures of the crossmodal integration responsible for the RHI.^24^^,^^56^ The task measures the interference of visual stimuli presented on the rubber hand on participants’ performance in a tactile discrimination task. Second, the Onset Time (OT) of the RHI has been indicated as a good predictor of the strength of the RHI.^23^ Finally, Subjective Reports (SR) of RHI are commonly considered a valid measure of participants’ phenomenology.^22^ Proprioceptive drift, another classical measure of RHI,^25^^,^^47^ was difficult to implement, as our eye-tracking montage would have interfered with the pointing procedure. Other measures of RHI, such as galvanic skin conductance response to a threating stimulus and skin temperature were also discarded as they were difficult to integrate within our setup/procedure.

#### Procedure

Each of the four experimental conditions (hand/eye movements – spatially congruent/incongruent) was tested in a separate block. Each block was divided in three phases (see below and Figure S1B). Appropriate training for each of the three tasks was provided before the beginning of the experiment. The training ensured that the participants were able to produce accurate hand- and eye-driven self-touch movements in our setup and to perform well all the tasks in our experiment. Eye tracking calibration was performed at the beginning of each block.

##### Phase 1. induction of the RHI

An example trial of this phase can be found in the Video S2. At the beginning of each trial a 15 × 15 mm blue square was projected on the easel, 20 cm to the right of the rubber hand. Participants were asked to look at the blue square to start the task. In both the hand and the eye movement conditions, participants were asked to gaze at the blue square to start a trial. Once gazed, the blue square turned green, after a short delay, prompting participants to start a hand or an eye movement towards the rubber hand. In the hand movement condition, participants used their right index finger to gently tap on the green square (starting point) and then on the Velcro band on the index finger of the rubber hand (landing point). Participants laid their right arm on an articulated armrest support throughout, so to produce straight movements from the starting to the landing points.

In the eye movement condition, participants rested their right hand out of sight and instead moved their gaze alternatively from the green square (starting point) to the Velcro band on the index finger of the cosmetic glove (landing point). Given that in the hand movement condition vision of the moving hand was necessary to perform the task, in the eye movement condition participants’ gaze was continuously shown by a 15 mm-diameter red circle projected on the easel. The visual cue thus ensured that the hand/eye movement conditions were comparable in terms of visual inputs (see Figure S5).

When a hand or an eye movement landed on the index finger of the rubber hand, participants received a simultaneous tactile stimulus on either their left index (spatially congruent condition) or little finger (spatially incongruent condition). The tactile stimuli in the hand movement condition were a direct replication of the signal recorded by the accelerometer, while the touches during the eye movement condition consisted of a playback of a pre-recorded signal, and was triggered by the participants’ gaze crossing a threshold on the right of the landing point. Using a rightward threshold rather than the precise landing point allowed us to compensate for the technical delay between the eye-landing on the target point and the tactile feedback (see Figure S4). The relative distance between the threshold and the landing point was determined in an informal pilot study where the participants adjusted the position of the threshold until their eye-landing on the target point of the rubber hand and the tactile feedback felt synchronous. The intensity of the pre-recorded tactile stimulus was jittered (by multiplying the amplitude of the signal pre-recorded via an accelerometer by a factor between 0.1 and 0.3). The jittered intensity was implemented in order to match the slight intensity variability of a touch induced by hand movements. In the spatially congruent condition, tapping/gazing on the index finger of the rubber hand produced a touch on the homologous finger of the participant, while in the spatially incongruent condition, tapping/gazing on the index finger of the rubber hand produced a touch on the little finger of the participant. A new trial started 500 ms after the tactile stimulation. Participants performed a total of 60 eye/finger movements in each condition, corresponding to about two minutes of stimulation.

An estimate of the Onset Time (OT) of the illusion^23^ was obtained in this phase. Participants were instructed to “step on a pedal as soon as the rubber hand feels part of yourself”. They were also told that this may or may not happen in different conditions.

##### Phase 2. crossmodal congruency task (CCT)

Immediately after the RHI induction phase, participants performed a tactile discrimination task adapted from the Crossmodal Congruency Task described by Zopf and colleagues.^24^ In each trial, a fixation cross was projected between the index and middle fingers of the rubber hand for 1.5 s. Then, a 1 cm-diameter yellow circle was projected on either the index or the middle finger of the rubber hand, flickering for 200 ms. After a jittered delay between 100 and 200 ms, a vibrotactile stimulus was delivered on the participants’ left hand. The tactile stimulus consisted of a series of three short pulses (total duration: 100 ms) delivered either on the homologous or non-homologous finger, with respect to the visual stimulus. Participants were asked to ignore the visual distractors and to respond as quick and as accurately as possible to the tactile stimulation only, by clicking on the left or right button of the mouse, according to the location of the touch. Response mappings was fixed across participants^24^ and required a left-click for a tactile stimulation on the middle finger and a right-click for a touch on the index finger. Feedback for correct, incorrect, or too-slow (i.e., ≥1.5 s) responses was provided after each trial.

To maintain the specific RHI conditions of a given block throughout the CCT task, after each CCT trial, participants were required to perform two further hand/eye movements from the crossmodal stimulation of that block.^24^^,^^52^ The CCT consisted of 30 homologous and 30 non-homologous trials presented in a fully randomised order. To ensure that participants looked at the visual stimuli, eight no-go trials were randomly presented during each block.^24^ In these trials, visual stimuli were delivered on both fingers of the rubber hand, but only one finger of the participants’ left hand was stimulated with a vibrotactile stimulus. Participants were instructed to withhold their response in these trials. Participants who responded to more than 35% of these trials were excluded from further analyses (two participants met this condition; see participants section).

##### Phase 3. subjective report (SR) of the RHI

We used two similar questionnaires to quantify participants’ phenomenological experience of the RHI induced by either hand or eye movements. The questionnaires were completed at the end of each block. Each questionnaire was composed by eight items directly derived from previous RHI studies.^22^ The eight items were divided in two categories: three experimental questions, aiming to quantify the strength of the illusion, and five control questions, aiming to assess participants’ suggestionability:

1. It seemed as if I were feeling the touch of my finger/the red light in the location where I touched/gazed the rubber hand.
2. It seemed as if the touch I felt was caused by my finger/the red light touching the rubber hand.
3. I felt as if the rubber hand were my hand.
4. It felt as if my (real) hand were drifting towards the rubber hand.
5. It seemed as if I might have more than one left hand.
6. It seemed as if the touch I was feeling came from somewhere between my own hand and the rubber hand.
7. It felt as if my (real) hand were turning ‘rubbery’.
8. It felt as if the rubber hand were drifting towards my (real) hand.

Each statement was projected directly onto the easel in front of the participants, in a randomised order. A Visual Analog Scale (VAS) from “strongly disagree” to “strongly agree” was presented below each statement, and participants used a mouse to adjust a slider according to their agreement with each sentence.

### Quantification and statistical analysis

All data met the normality assumption (p>.057 in all cases), therefore, parametric tests were used throughout (see Data S1). Analysis for the CCT followed Zopf et al.^24^ Trials in which participants responded too soon (≤150 ms) or too late (≥1500 ms) were discarded from the analyses and only the reaction times (RT) for correct responses were used.^24^ The CCT effect was calculated as participants’ performance on non-homologous minus homologous trials.^24^ As accuracy and RT in the CCT are interdependent,^24^^,^^57^ results from each participant were integrated into an Inverse Effectiveness Score (CCT-IES; reported in Figure 1C in the main text).^24^^,^^57^^,^^58^ Supplemental analyses of participants’ reaction times (CCT-RT) and percentage errors (CCT-PE) showed substantially similar results than the combined CCT-IES measure (see Figure S2). A 2 (effector-type: hand, eye) x 2 (spatial-congruency: congruent, incongruent) repeated measures ANOVA was used to analyse CCT data.

The Onset Time of the RHI was analysed with a similar 2 × 2 rmANOVA. Yet, given that not all participants reported an illusion in all four experimental conditions, statistical analyses were only run on the data from participants who responded in all the blocks (n = 12; Figure 1D). The rationale for this choice is that missing values may potentially be due to a range of reasons, from not experiencing any RHI to forgetting the instructions. For example, during the debriefing at the end of the experiment a few participants even reported they had forgotten to step on the pedal *because* they were experiencing a strong RHI (see Figure S3 for details on formal analyses supporting this rationale).

Finally, given that Subjective Reports were given on a continuous visual analog scale (from −100 = strongly disagree to +100 = strongly agree) instead of a 7-points Likert scale,^22^ participants’ responses were also analysed through parametric tests. Participants’ agreement with each statement of the questionnaire were first averaged across question type (i.e., RHI/control items), and then analysed in a 2 (spatial-congruency: congruent, incongruent) x 2 (effector-type: hand, eye) x 2 (item-type: illusion, control) rmANOVA.

For each of the three dependent variables, we expected a main effect of spatial-congruency, in line with the general principle that crossmodal stimulation must be spatially congruent in order to elicit the RHI.^37^ Crucially, in line with our main hypothesis of similar RHI for hand and eye movements, we also predicted no significant interaction between main effects. Given that the experiment was designed to allow asserting the null hypothesis (i.e., eye movements and hand movements induce the same amount of RHI), non-significant interactions between spatial-congruency and effector-type were further tested through Bayesian t-tests on the difference between spatially congruent/incongruent conditions in each type of movement. This allowed us to determine whether the datasupported the null hypothesis or if, alternatively, the null result could reflect insufficient statistical power.^26^ All the ANOVAs were conducted using IBM SPSS Statistics for Windows, version 23 (IBM Corp.,USA). Bayesian analyses were run on JASP v. 0.12.1 (JASP Team 2016, University of Amsterdam).

## Data Availability

All data needed to evaluate the conclusions in the paper are present in the paper and/or the supplemental information. This paper does not report original code. Any additional information is available from the lead contact upon request.

## Supplemental information

Document S1. Figures S1–S5

Data S1. (Separate file) All data necessary for the analyses reported in this study, Related to Figures 2
