# Supplementary material for: Touching with the eyes: Oculomotor self-touch induces illusory body ownership
Source: iScience. 2023 Feb 10;26(3):106180. doi: 10.1016/j.isci.2023.106180 (PMC9988563; doi:10.1016/j.isci.2023.106180)
Supplement: Document S1. Figures S1–S5 [file mmc1.pdf]

iScience, Volume *26*

Supplemental information

Touching with the eyes: Oculomotor self-touch induces illusory body ownership

Antonio Cataldo, Massimiliano Di Luca, Ophelia Deroy, and Vincent Hayward

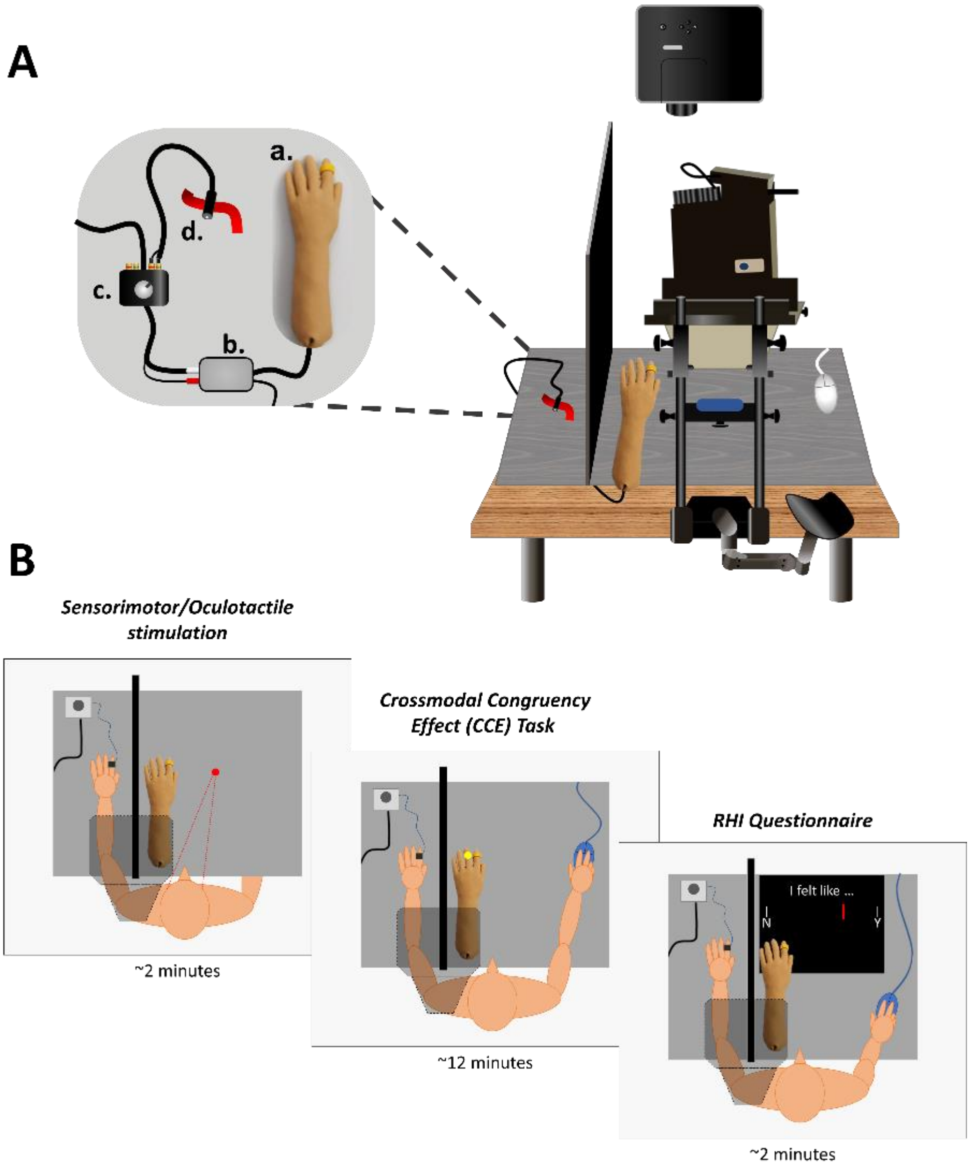

Figure S1, Related to Figure 1. A. Experimental setup. View from the participants' vantage point of the desk and apparatus. The participant's left arm was positioned behind the occluder, and the right arm rested on the articulated armrest. Eye movements were recorded by an eye-tracker in a tower-mount configuration and participants could see the setup through a transparent 45° hot mirror. Instructions and visual stimuli were presented via a downward-facing LCD projector installed above the desk. The setup for the "haptic" RHI (left panel) involved an accelerometer located inside the index finger of the cosmetic glove (a.), a custom-made computer-controlled switch (b.), an amplifier (c.), and three vibrotactile actuators (only one shown here for clarity, d.). In the hand-driven "proprioception" movement RHI condition, participants used their right index finger to tap on the index finger of the rubber hand, andhand and received a synchronous touch on either their left index or little finger. In the eye-driven "no-proprioception" movement RHIcondition, participants' gaze to the index finger of the rubber hand produced a tactile

feedback on either the participants' left index or little finger. **B. Experimental procedure.** The experiment consisted of three phases. First, in a RHI induction phase, participants performed sixty hand/eye movements toward the rubber hand. Each movement generated spatially congruent or incongruent tactile sensations on the participant left hand. During this phase, participants also reported the onset time (OT) of the illusion. Immediately after the RHI induction phase, participants performed a crossmodal congruency task (CCT). Finally, participants answered to a brief subjective report (SR) questionnaire aiming to capture their phenomenological experience during the crossmodal stimulation.

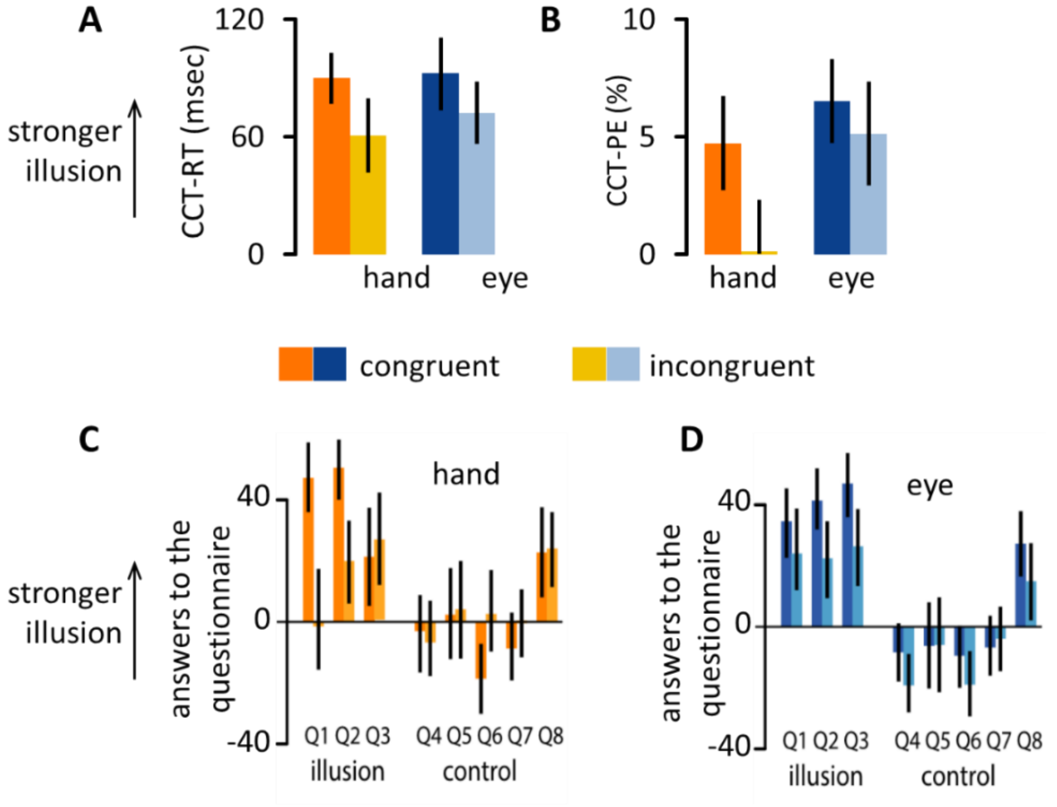

Figure S2, Related to Figure 2. A. CCT-RT results. Participants' reaction times to homologous and non-homologous trials in the CCT produced similar results to the CCT inverse effectiveness score reported in the main text. In particular, a supplemental rmANOVA showed a significant main effect of spatial-congruency ($F(1, 23) = 5.223$, $p = 0.032$, pη2 = 0.185), but no effect of effector-type ($F(1, 23) = 0.398$, $p = 0.534$, pη2 = 0.017), nor interaction between the main effects ($F(1, 23) = 0.135$, $p = 0.717$, pη2 = 0.006). A Bayesian t-test supported the null interaction (BF01 = 4.380, error = 0.034%). **B. CCT-PE.** Differences in the percentage error data, instead, did not reach statistical significance (spatial-congruency: $F(1, 23) = 1.947$, $p = 0.1761$, pη2 = 0.078; effector-type: $F(1, 23) = 3.878$, $p = 0.061$, pη2 = 0.144; interaction: $F(1, 23) = 1.080$, $p = 0.310$, pη2 = 0.045), but, overall, the number of percentage errors was numerically higher (5.6 ± 8.5 %) in the spatially congruent condition, compared to the spatially incongruent condition (2.6 ± 8.1), as expected. **C-D. Subjective Report results.** Participants' subjective reports on the illusion and control items of the RHI questionnaire were similar across hand (**C**) and eye (**D**) movement conditions. In particular, a 2 (spatial-congruency: congruent, incongruent) x 2 (effector-type: hand, eye) x 2 (item-type: illusion, control) rmANOVA showed a significant main effect of spatial-congruency ($F(1, 19) = 4.631$, $p = 0.044$, pη2 = 0.196) and item-type ($F(1, 19) = 24.127$, $p < 0.001$, pη2 = 0.559), and a significant interaction between the two factors ($F(1, 19) = 11.547$, $p = 0.003$, pη2 = 0.378). Overall, participants' ratings were significantly higher for the experimental (32.3 ± 13.3) compared with the control (-1.0 ± 15.4) items, and for the spatially congruent (20.6 ± 12.7) compared with the spatially

incongruent stimulation (10.7 ± 14.1). Importantly, the main effect of effector-type and all the remaining interactions were not statistically significant ($p > 0.07$ in all cases), with participants giving similar scores to the eye and finger movement conditions. The null interaction between effector-type and spatial-congruency ($F(1, 19) < 0.001$, $p = 0.991$, $p\eta 2 < 0.001$) was confirmed by a paired sample Bayesian t-test (BF01 = 3.1, error = 0.020 %), showing that the data were three times more likely under the null than the alternative hypothesis. Error bars thrugout the figure show the SEM.

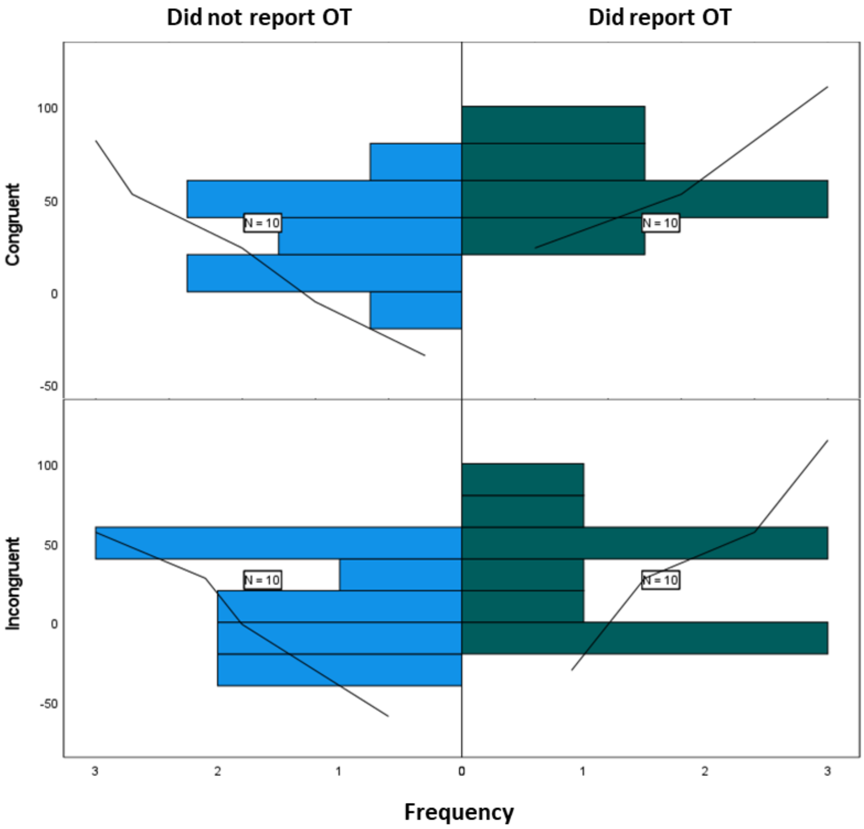

Figure S3, Related to Figure 2. Supplemental analysis on omitted Onset Time data. The frequency of missing OT responses in each condition was: eye-congruent = 25%; eye-incongruent = 45.8; hand-congruent = 41.7%; hand-incongruent = 33.3. A series of pairwise McNemar's tests showed that the number of missing values was not statistically different between conditions ($p \geq 0.063$ in all cases). To test whether the omitted OT responses were exclusively due to a lack of RHI or could instead reflect random factors, we analysed the distribution of self-report questionnaire data of the participants who did and did not report the OT in all blocks. In particular, we averaged participants' responses to the illusion items across effector type conditions and ran two independent-sample Kolmogorov-Smirnov tests on the congruent and incongruent conditions. If omitted OT univocally reflected lack of RHI, then one would expect a significantly different (i.e. right-skewed) distribution for those participants, compared to the participants who did report the OT. Contrary to this prediction, the distribution of reported strength of the illusion was overall very similar between the participants who did and did not report the OT (congruent condition: $D(20) = 1.118$, $p = .164$; incongruent condition: $D(20) = 0.671$, $p = .759$), suggesting that it would be wrong to interpret the missing OT values as an absence of RHI.

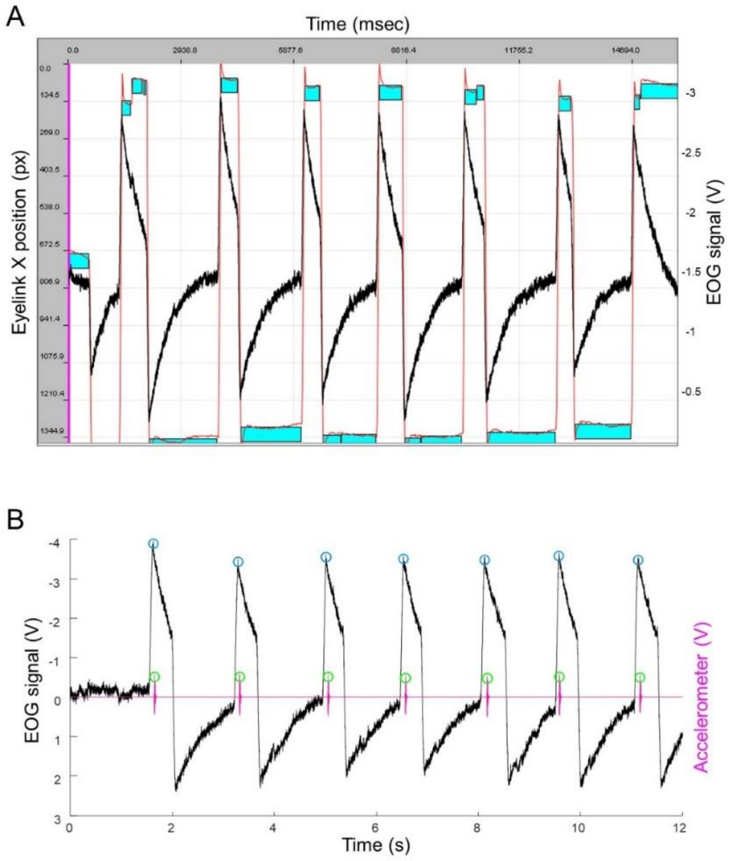

Figure S4, Related to Figure 1. A. EOG validation of our eye-tracking system. Delays between the fixation at the target point and the delivery of the tactile stimulus in our system can theoretically arise from three possible sources: eye-tracker, experiment software, and audio buffer transduction. We tested the delay in our eye-tracking system using electrooculography (EOG) to record the electrical signal from the lateral rectus muscles of the eyes. Although EOG is not very accurate in the spatial domain (~1° resolution), this technique has a very high temporal resolution, providing an optimal independent measure of the saccade onset and end. The results showed that the signal from the EOG signals (black traces superimposed in panel **A**) was highly correlated to our Eyelink 100 plus, with negligible difference between the two systems. **B. Delay between end of saccade and touch.** Next, we simultaneously recorded EOG data and the signal from an accelerometer fixed to our tactile actuator. This allowed us to measure the precise delay between the end of an eye movement and the onset of a tactile stimulus. The results showed that this delay was relatively small and systematic (mean ± SD: 33.14 ± 13.66 ms). In our experiment, we compensated for this technical delay by using an arbitrary threshold to the right of the target point to trigger the gaze-induced touches. The relative distance between the threshold and the landing point was determined in an informal pilot study where the participants adjusted the position of the threshold until their eye-landing on the target point of the rubber hand and the tactile feedback felt synchronous.

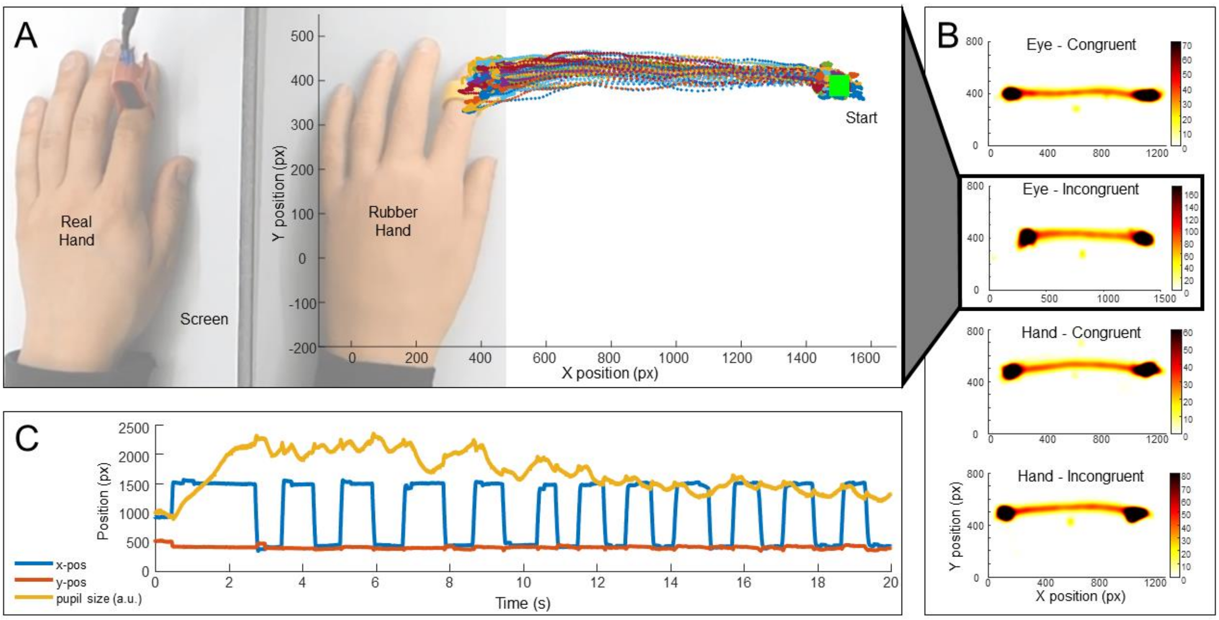

Figure S5, Related to Figure 1. A. Traces of 60 eye movements in the stimulation phase of block #2 (Eye – Incongruent) in a representative participant (P #2). We recorded the participants' gaze in the stimulation phase of all four experimental conditions. In both the hand and the eye movement conditions, participants were asked to gaze at a blue square to the right of the rubber hand (start point) to start a trial. Once gazed, the blue square turned green after a short delay, prompting participants to start a hand or an eye movement towards the rubber hand and to fixate the landing point on the index finger of the rubber hand. Different colours show the trajectory of different leftward saccades in different trials. The position of the hands and green square is for illustrative purposes only, as the position of the different items is not calibrated with respect to the data. **B. Heatmaps of eye movements in all conditions.** The procedure described above provided a consistent pace of stimulation across all conditions, and ensured that the participants' eye movements were very similar across all conditions. **C. Continuous eye-tracking data.** The graph shows the traces of X and Y position (in pixels) and pupil size (a.u.) over time for the first 20 s of the stimulation phase in block #2 for participant #2.
